# Survival outcomes and influencing factors in Zhengzhou HIV/AIDS patients following antiretroviral therapy initiation (2014-2024): a retrospective cohort analysis

**DOI:** 10.3389/fpubh.2026.1733724

**Published:** 2026-03-11

**Authors:** Meng Deng, Yan Sun, Xuan Yang, Qiong Li, Zhihui Zhang, Zhaoyun Chen

**Affiliations:** 1 Clinical Medical Research Center, Henan Infectious Diseases Hospital, Zhengzhou, China; 2 Clinic of Infectious Diseases and Clinical Immunology, Henan Infectious Diseases Hospital, Zhengzhou, China; 3 Department of Infectious Diseases, Henan Infectious Diseases Hospital, Zhengzhou, China

**Keywords:** antiretroviral therapy, COX proportional hazards regression model, human immunodeficiency virus, influencing factors, survival analysis

## Abstract

**Background:**

While antiretroviral therapy (ART) has significantly improved the long-term survival of people living with HIV/AIDS, leading to a chronic disease management model, survival outcomes can be influenced by demographic and clinical factors. There is a need to evaluate the long-term survival of HIV-infected individuals initiating ART and identify local influencing factors to optimize patient management and improve prognosis.

**Methods:**

A retrospective cohort study was conducted on HIV-infected individuals who initiated ART in Zhengzhou between 2014 and 2024. Demographic data and ART-related information were collected from the National AIDS Clinical Data System. The life table method was employed to describe patient survival time, while the Kaplan-Meier method was used to compare survival time differences under various conditions and to plot survival curves. A Cox proportional hazards regression model was applied to analyze risk factors influencing patient survival time.

**Results:**

Among the 3,312 HIV-infected individuals, the total follow-up time amounted to 15,656.5 person-years, with a median follow-up of 4.73 years. A total of 107 deaths were recorded, yielding a mortality rate of 0.68 per 100 person-years. The cumulative survival rates at 1, 3, 5, and 10 years were 99%, 98%, 96%, and 93%, respectively. Multivariate Cox regression analysis identified age greater than 60 years (HR = 5.570, 95% CI: 1.608–19.292) as a risk factor for mortality. Additionally, patients with a baseline CD4^+^ T lymphocyte count of less than 50 cells/μL faced a significantly higher risk of death compared to those with a count greater than 350 cells/μL (HR = 3.777, 95% CI: 1.583–9.014).

**Conclusion:**

From 2014 to 2024, the overall survival of HIV-infected individuals receiving antiviral therapy in Zhengzhou was favorable. However, advanced age and a low baseline CD4^+^ T lymphocyte count were identified as significant factors for an elevated mortality risk. It is recommended to enhance clinical management for older patients and to initiate treatment early to improve CD4^+^ T lymphocyte levels, thereby further improving survival outcomes.

## 1 Introduction

Human Immunodeficiency Virus (HIV) infection and its progression to Acquired Immune Deficiency Syndrome (AIDS) remain a critical global public health issue (1). By the end of 2022, an estimated 39.9 million individuals were living with HIV worldwide, with approximately 630,000 AIDS-related deaths recorded in that year alone (2). In China, cumulative reported HIV cases reached 1.223 million by the same time, alongside 418,000 cumulative deaths (3). The advent and optimization of antiretroviral therapy (ART), supported by standardized clinical management and evolving treatment protocols, have fundamentally transformed HIV/AIDS from a rapidly fatal condition into a manageable chronic disease. This therapeutic progress has substantially reduced both AIDS-related morbidity and mortality (4).

In alignment with China's “Healthy China” strategy, which emphasizes whole-life-cycle health management, the focus of AIDS prevention and control is now shifting from merely ensuring effective treatment to promoting quality survival. This shift aligns with the changing demographics of China's HIV population, which is increasingly characterized by aging, extended survival, and significant heterogeneity in health outcomes. Survival among people living with HIV is influenced not only by ART but also by a range of sociodemographic, clinical, and immunological factors, including age, education, transmission route, baseline immune status, and disease stage at diagnosis (5, 6). These factors collectively contribute to considerable variation in long-term prognosis, highlighting the need for multidimensional approaches to care.

As one of China's most populous provinces, Henan represents a critical region for HIV/AIDS prevention and control. Zhengzhou, its provincial capital, has achieved early and widespread ART coverage, resulting in a substantial and well-established cohort of long-term survivors (7). This offers a valuable opportunity for in-depth survival analysis in a representative urban setting. However, most existing studies either focus on other regions or are limited by short follow-up periods, leaving a gap in evidence that reflects the unique epidemiological and public health context of Zhengzhou as a major central Chinese city.

In the context of transitioning from ensuring survival to promoting quality of life, there is an urgent need to systematically evaluate long-term survival outcomes and their determinants among HIV/AIDS patients in Zhengzhou. This study therefore adopts a retrospective cohort design to analyze survival rates and identify prognostic factors in individuals receiving ART in Zhengzhou from 2014 to 2024. The results aim to provide localized evidence to guide tailored clinical interventions, support precision in patient management, and ultimately contribute to extended survival and enhanced quality of life for people living with HIV in this region.

## 2 Materials and methods

### 2.1 Participants

This study was conducted among HIV-infected individuals in Zhengzhou City. Data were sourced from the AIDS Clinical Diagnosis and Treatment Data Collection System, covering the period from January 1, 2014, to December 31, 2024. The system has achieved regular data synchronization with the comprehensive surveillance network of the Henan Provincial Center for Disease Control and Prevention (which includes official death registration information), thereby capturing and supplementing death events that did not occur within the treatment facilities but were registered at the place of household registration. This effectively minimizes underreporting caused by loss to follow-up or out-of-hospital deaths. The inclusion criteria were as follows: (1) confirmed HIV antibody positivity by Western Blot (WB) assay; (2) initiation of antiretroviral therapy (ART) for the first time between 2014 and 2024. Exclusion criteria included: (1) individuals currently residing in other provinces or foreign nationals; (2) cases with severely missing baseline or follow-up information.

### 2.2 Study design

This study defined all-cause mortality as the endpoint event. Survival time was calculated as the interval from the initiation of ART until the occurrence of the endpoint event (death of the participant), a censoring event (including patients still on treatment, lost to follow-up, transferred to other medical institutions, or treatment discontinuation as of the end of the observation period), or the study cutoff date (December 31, 2024). Definition of Loss to Follow-up: no system records for more than 12 months after the last documented visit and no confirmation of death.

The collected data include:

(1) Demographic characteristics: sex, age, marital status, etc.
(2) Clinical and infection-related information: HIV diagnosis date, transmission route, baseline CD4^+^ T lymphocyte count, baseline HIV viral load, baseline WHO clinical stage, ART Regimens etc.
(3) Outcome-related information: date of death and cause of death.

Routine patient follow-up was performed by clinical and public health staff adhering to national protocols, entailing quarterly reviews in the first 6 months post-ART initiation, followed by semi-annual assessments that incorporated symptom checks, CD4^+^ T lymphocyte count, and viral load monitoring.

### 2.3 Laboratory tests

HIV Viral Load: Measured by real-time quantitative PCR technology using the Abbott m2000sp system (Abbott Laboratories, USA) with Abbott original reagent kits, with a lower limit of detection of 40 copies/ml.

CD4^+^ T Lymphocyte Count: Measured by flow cytometry using the BD FACSCalibur instrument NAVIOS (BD Biosciences, USA) with original BD reagent kits.

### 2.4 Statistical analysis

Statistical analyses were performed with IBM SPSS Statistics (Version 26.0). Categorical variables are expressed as frequencies and percentages, and group comparisons were made using the Chi-square test or Fisher's exact test, as appropriate. The normality of continuous variables was assessed, and non-normally distributed data are summarized as the median and interquartile range (IQR).

Survival analysis was conducted using the Kaplan-Meier method, and survival curves between groups were compared with the log-rank test. Before performing multivariate analysis, multicollinearity among potential predictors was evaluated by calculating the variance inflation factor (VIF). A VIF threshold of five was applied, whereby variables with VIF ≥5 were considered to exhibit significant multicollinearity and were excluded from subsequent models. All retained variables had VIF values below five, indicating acceptable collinearity.

Predictors of therapy modification were identified using Cox proportional hazards regression. Results are reported as adjusted hazard ratios (AHR) with 95% confidence intervals (CI). A two-sided *p*-value < 0.05 was considered statistically significant for all tests.

## 3 Results

### 3.1 Baseline characteristics

A total of 3,346 medical records of HIV/AIDS patients were initially collected based on the inclusion criteria. After excluding 34 cases with severely incomplete follow-up information, the final study population comprised 3,312 individuals. Among them, 3,054 (92.21%) were male and 258 (7.79%) were female. The mean age was 42.19 ± 14.25 years. The majorities were unmarried (52.42%), and the predominant route of HIV transmission was homosexual contact (64.31%). At ART initiation, 2,548 patients (76.87%) were at WHO clinical stage I. A total of 1,062 individuals (32.07%) had a baseline CD4^+^ T lymphocyte count greater than 350 cells/μL, while 891 (26.90%) had a baseline HIV viral load exceeding 50,000 copies/ml. Two thousand, five hundred seventeen patients (76.00%) initiated treatment with a non-nucleoside-based regimen. Other detailed baseline characteristics are presented in Table 1.

**Table 1:** Demographic characteristics of HIV/AIDS patients on ART in Zhengzhou.

**Variables**	**Number of cases (proportion, %)**	**Mortality, % (*n*)**	***X* ^2^**	***p* value**
**Gender**				
Male	3,054 (92.21)	3.11 (95)	1.804	0.179
Female	258 (7.79)	4.65 (12)		
**Age (years)**				
< 30	612 (18.48)	1.31 (8)	214.710	< 0.001
31–45	1,601 (48.34)	0.75 (12)		
46–59	685 (20.68)	3.8 (26)		
≥60	414 (12.50)	14.73 (61)		
**Marital status**			44.985	< 0.001
Never married	1,736 (52.42)	1.27 (22)		
Married	1,242 (37.50)	5.39 (67)		
Divorced/widowed	334 (10.08)	5.39 (18)		
**Infection route**			30.296	< 0.001
Homosexual	2,130 (64.31)	1.97 (42)		
Heterosexual	1,097 (33.12)	5.50 (60)		
Blood-borne	85 (2.57)	5.88 (5)		
**WHO clinical staging at ART initiation**			11.152	0.011
I	2,548 (76.93)	2.75 (70)		
II	192 (5.80)	4.17 (8)		
III	87 (2.63)	2.30 (2)		
IV	485 (14.64)	5.57 (27)		
**Baseline CD4**^+^ **T cell count(cells/**μ**L)**			42.331	< 0.001
< 50	323 (9.75)	8.97 (29)		
50–200	738 (22.28)	3.79 (28)		
200–350	1,189 (35.90)	2.10 (25)		
>350	1,062 (32.07)	2.35 (25)		
**Baseline HIV RNA(copies/ml)**			7.830	0.020
< 10,000	956 (28.85)	1.78 (17)		
10,000–50,000	770 (23.25)	2.08 (16)		
>50,000	891 (26.90)	3.70 (33)		
**ART regimens**			10.706	0.005
Non-nucleoside reverse transcriptase inhibitors	2,517 (76.00)	3.73 (94)		
Protease inhibitors	97 (2.93)	4.12 (4)		
Integrase inhibitors	698 (21.07)	1.29 (9)		
**Time interval between HIV/AIDS diagnosis and ART initiation**			3.687	0.158
< 1 weeks	712 (21.50)	2.11 (15)		
1 weeks−6 months	2,190 (66.12)	3.52 (77)		
>6 months	410 (12.38)	3.66 (15)		

### 3.2 Mortality outcomes

A total of 3,312 patients receiving ART were followed for a cumulative 15,656.5 person-years, with a mean follow-up duration of 4.73 person-years. By the end of December 31, 2024, 107 deaths were recorded, corresponding to a mortality rate of 0.68 per 100 person-years.

Analysis revealed a higher mortality rate among females (4.65%) compared to males (3.11%). Patients aged 60 years or older exhibited the highest mortality rate (14.73%), while married individuals had a mortality rate of 5.39%. In terms of transmission routes, the mortality rate was lowest among those infected via homosexual contact (1.97%), compared to 5.50% for heterosexual transmission and 5.88% for blood-borne transmission. Additionally, patients initially diagnosed at WHO clinical stage IV had a mortality rate of 5.57%. Regarding baseline immunologic and virologic status, the mortality rate was 8.97% among those with a baseline CD4^+^ T lymphocyte count < 50 cells/μL, and 3.70% for those with a baseline HIV RNA ≥50,000 copies/ml. Patients who initiated ART more than 6 months after diagnosis had a mortality rate of 3.66%.

### 3.3 Survival outcomes

**Figure 1:**
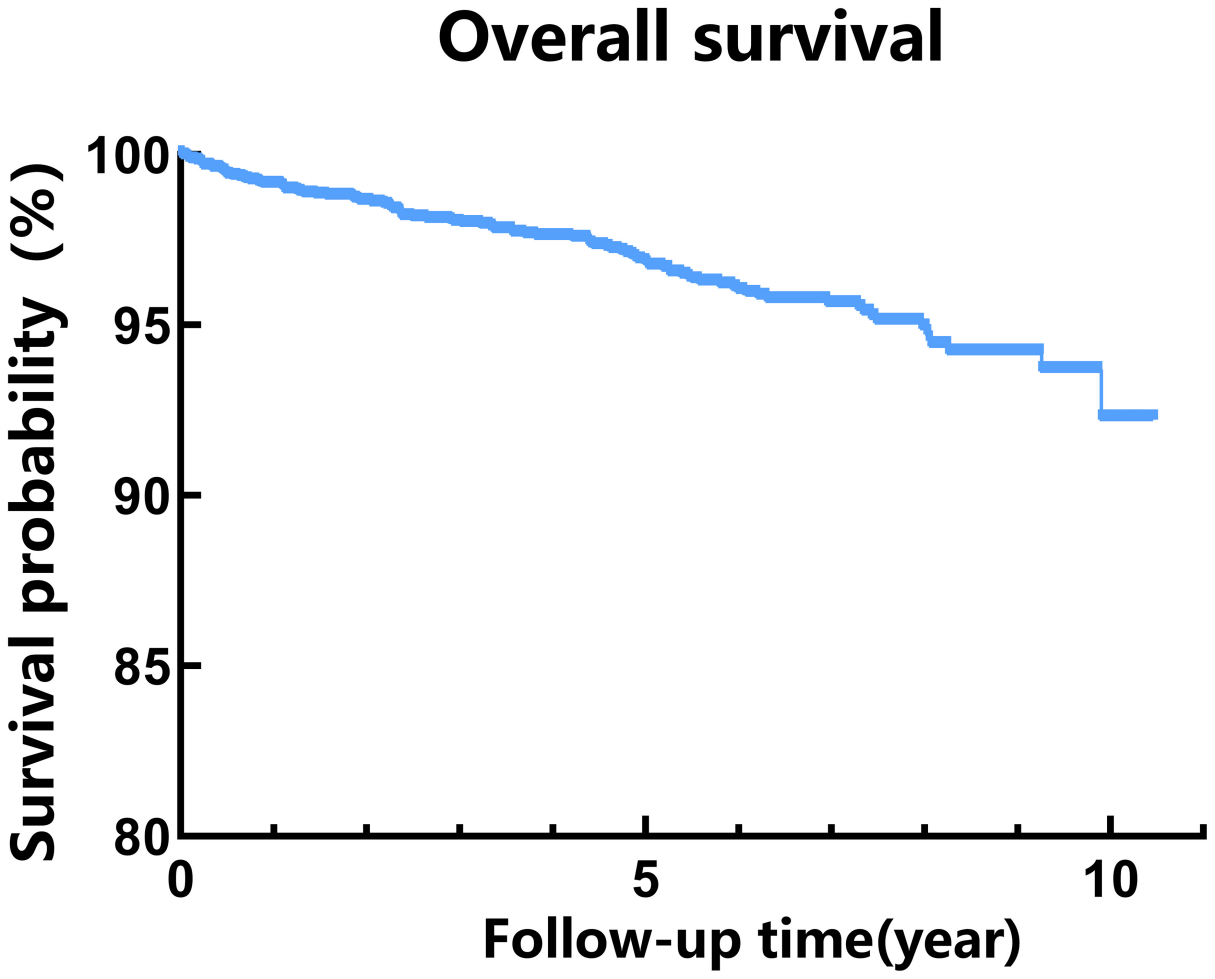
Survival curve for patients receiving ART in Zhengzhou.

The median survival time of HIV patients receiving ART was 4.76 (2.19, 7.06) years. The cumulative survival rates at 1, 3, 5, and 10 years post-ART initiation were 99%, 98%, 96%, and 93%, respectively. Detailed results are presented in Table 2 and Figure 1.

**Table 2:** Cumulative survival rate of HIV/AIDS patients receiving ART in Zhengzhou.

**Time interval (Year)**	**Number entering interval**	**Censored data**	**Number exposed to risk**	**Number of deaths (*n*)**	**Proportion surviving, %**	**Cumulative survival rate,%**	**Cumulative survival rate standard error**
**0~**	3,312	437	3,093.50	29	0.99	0.99	0.00
**1~**	2,846	294	2,699.00	14	0.99	0.99	0.00
**2~**	2,538	273	2,401.50	15	0.99	0.98	0.00
**3~**	2,250	332	2,084.00	9	1.00	0.98	0.00
**4~**	1,909	324	1,747.00	13	0.99	0.97	0.00
**5~**	1,572	406	1,369.00	12	0.99	0.96	0.00
**6~**	1,154	303	1,002.50	4	1.00	0.96	0.00
**7~**	847	290	702.00	5	0.99	0.95	0.01
**8~**	552	313	395.50	4	0.99	0.94	0.01
**9~**	235	189	140.50	2	0.99	0.93	0.01
**10~**	44	44	22.00	0	1.00	0.93	0.01

### 3.4 Comparison of cumulative survival rates

Kaplan-Meier analysis with log-rank tests revealed statistically significant differences in cumulative survival rates across several baseline characteristics, including age, marital status, transmission route, WHO clinical stage, baseline CD4^+^ T lymphocyte count, and baseline HIV RNA level.

**Figure 2:**
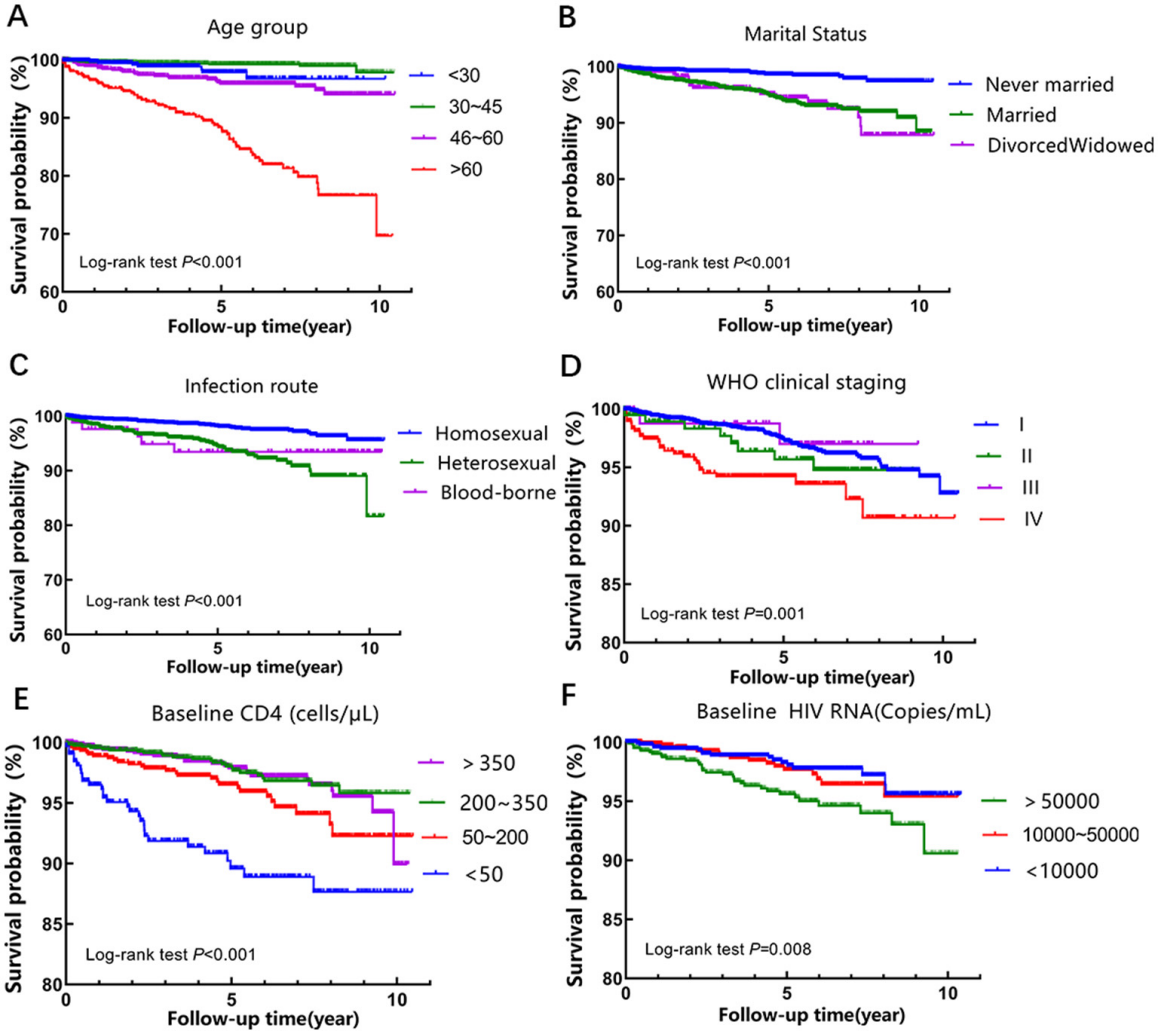
Kaplan-Meier survival curves for intergroup differences according to **(A)** age at diagnosis, **(B)** marital status, **(C)** infection route, **(D)** WHO clinical stage, **(E)** baseline CD4^+^ T cell count, and **(F)** baseline HIV RNA.

Specifically, patients aged ≥60 years at baseline had the lowest cumulative survival rate compared to other age groups (χ^2^ = 187.207, *P* < 0.001). Unmarried patients exhibited a higher cumulative survival rate than those who were married, divorced, or widowed (χ^2^ = 36.825, *P* < 0.001). In terms of transmission routes, homosexual contact had a higher cumulative survival rate compared to other transmission categories (χ^2^ = 34.736, *P* < 0.001). Furthermore, significantly lower cumulative survival rates were observed in patients who were at WHO clinical stage IV (χ^2^ = 16.894, *P* = 0.001), had a CD4^+^ T lymphocyte count < 50 cells/μL (χ^2^ = 43.160, *P* < 0.001), or had an HIV RNA level > 50,000 copies/ml (χ^2^ = 9.556, *P* = 0.008). The corresponding survival curves are shown in Figure 2.

### 3.5 Analysis of factors influencing survival time

Univariate Cox proportional hazards regression analysis indicated that age at confirmed diagnosis, marital status, disease stage at diagnosis, transmission route, initial CD4^+^ count, ART Regimens and baseline HIV RNA level were significant factors influencing survival among HIV/AIDS patients. Variables demonstrating statistical significance (*P* < 0.05) in the univariate analysis were subsequently included in the multivariate Cox model. The results identified the following independent predictors of mortality: age greater than 60 years (HR = 5.570, 95% CI:1.608~19.292) and a baseline CD4^+^ T lymphocyte count of less than 50 cells/μL (HR = 3.777, 95% CI: 1.583~9.014), as shown in Table 3.

**Table 3:** Analysis of factors associated with mortality in HIV/AIDS patients in Zhengzhou.

**Variables**	**Univariate model**		**Multivariate model**	
	**Crude HR (95%** ***CI*****)**	***P*** **-value**	**Adjusted HR (95%** ***CI)***	***P*** **-value**
**Gender**				
Male	1.000		–	
Female	1.439 (0.789~2.624)	0.235		
**Age (years)**				
< 30	1.000		1.000	
31~45	0.384 (0.156~0.941)	0.036	0.231 (0.074~0.720)	0.012
46~59	1.787 (0.805~3.965)	0.154	1.274 (0.384~4.231)	0.692
≥60	7.351 (3.503~15.425)	< 0.001	5.570 (1.608~19.292)	0.007
**Marital status**				
Never married	1.000		1.000	
Married	3.788 (2.339~6.134)	< 0.001	0.851 (0.334~2.166)	0.735
Divorced/widowed	4.071 (2.183~7.591)	< 0.001	0.542 (0.178~1.652)	0.281
**Infection route**				
Homosexual	1.000		1.000	
Heterosexual	3.094 (2.083~4.596)	< 0.001	1.331 (0.772~2.294)	0.303
Blood-borne	2.167 (0.854~5.499)	0.104	0.989 (0.291~3.365)	0.986
**WHO clinical staging at ART initiation**				
I	1.000		1.000	
II	1.425 (0.683~2.974)	0.345	0.836 (0.296~2.361)	0.735
III	0.843 (0.206~3.444)	0.812	0.308 (0.041~2.258)	0.249
IV	2.452 (1.567~3.837)	< 0.001	0.896 (0.446~1.801)	0.758
**Baseline CD4**^+^ **T cell count(cells/**μ**L)**				
< 50	3.967 (2.323~6.773)	< 0.001	3.777 (1.583~9.014)	0.003
50~200	1.663 (0.970~2.853)	0.065	1.180 (0.556~2.501)	0.666
200~350	0.904 (0.519~1.573)	0.720	0.854(0.414~1.761)	0.668
>350	1.000		1.000	
**Baseline HIV RNA(copies/mL)**				
< 10,000	1.000		1.000	
10,000~50,000	1.173 (0.593~2.322)	0.646	1.102 (0.553~2.195)	0.783
>50,000	2.253 (1.255~4.045)	0.007	1.400 (0.738~2.657)	0.303
**ART regimens**			-	
Non-nucleoside reverse transcriptase inhibitors	1.000			
Protease inhibitors	1.109 (0.399~3.080)	0.843	0.899 (0.215~3.765)	0.884
Integrase inhibitors	0.337 (0.169~0.671)	0.002	0.799 (0.364~1.756)	0.577
**Time interval between HIV/AIDS diagnosis and ART initiation**				
< 1 weeks	1.000		-	
1 weeks~6 months	1.469 (0.845~2.556)	0.173		
>6 months	1.757 (0.858~3.596)	0.123		

Not included in the multivariate analysis.

## 4 Discussion

The survival status of HIV/AIDS patients following long-term ART has become an increasing focus of clinical research. Understanding the predictors of survival and mortality is critical for implementing corrective measures in routine clinical services and informing health policy development. Accordingly, this study focused on the survival status and influencing factors among HIV/AIDS patients receiving ART in Zhengzhou from 2014 to 2024. The results demonstrated a high overall survival rate in this retrospective study, with cumulative survival rates at 1, 3, 5, and 10 years reaching 99%, 98%, 96%, and 93%, respectively. These findings are consistent with recent reports from major domestic urban cohorts (e.g., Beijing, Tianjin, Shandong, Hangzhou) (8–11), collectively reflecting the long-term survival levels currently achieved by HIV-infected individuals under antiretroviral therapy in China. This may be attributed to the cohort's urban profile, widespread early diagnosis and treatment, broad use of optimized regimens, and enhanced regional healthcare services. In this study, several factors associated with mortality risk were identified, including age and CD4^+^ T lymphocyte count.

In recent years, the aging of the AIDS population has garnered global attention. UNAIDS estimates indicate that the number of HIV/AIDS patients aged 50 or older worldwide increased from 5.4 million in 2015 to 8.1 million in 2020 (12). Previous studies have confirmed that age is a key factor influencing the survival prognosis of HIV/AIDS patients (13). The underlying mechanism may involve the progressive damage to the immune system in older patients due to HIV infection, which exacerbates with age and significantly increases susceptibility to related complications. Concurrently, the accelerated shortening of telomere length in CD4^+^ T cells among elderly HIV/AIDS patients leads to reduced cellular replicative capacity. As a result, the rate of CD4^+^ T cell recovery is approximately 40% slower than in younger patients. Even with effective viral suppression, CD4^+^ counts often continue to decline, further impairing immune function (14). These factors collectively contribute to a significantly increased risk of mortality in the older population, with a clear upward trend in fatality rates among higher age groups (15). This study reached a similar conclusion, showing that the risk of death in patients over 60 years of age was 5.570 times higher than in those under 30. This finding underscores the need for enhanced treatment and efficacy monitoring for older adults living with HIV/AIDS in clinical practice to reduce AIDS-related mortality in this population.

CD4^+^ T lymphocyte count is a key indicator for assessing immune function in HIV/AIDS patients and has a significant impact on disease progression and mortality risk. Studies have confirmed that the recovery of immune function is an important factor in improving the survival prognosis of HIV/AIDS patients (16). As the most direct reflection of the body's immune status after HIV infection, a low CD4^+^ T lymphocyte count is significantly associated with an increased risk of opportunistic infections and elevates the mortality risk from AIDS-related diseases (17).

The results of this study indicate that patients with a baseline CD4^+^ T lymphocyte count below 50 cells/μL had a higher risk of death compared to those with a count above 350 cells/μL. This finding is consistent with the results reported by Yang et al. in their analysis of survival rates among long-term HIV/AIDS cases aged 15 and older receiving antiretroviral therapy in Henan Province (18). Similarly, Li et al. reported that patients with higher baseline CD4 counts had a greater probability of survival (19). Collectively, our findings reinforce the current World Health Organization guidelines, which recommend the prompt initiation of antiretroviral therapy for all people living with HIV, regardless of CD4^+^ T cell counts. Notably, the higher mortality observed among patients with CD4^+^ T cell counts < 50 cells/μL highlights the particular urgency of timely treatment initiation in this subgroup, along with the need for enhanced clinical vigilance to screen for and prevent opportunistic infections.

This study has several limitations. First, there was a certain proportion of loss to follow-up during the observation period, and those lost to follow-up were generally younger. Given that older age is a clearly identified risk factor for mortality in this study, this bias may lead to an overestimation of overall mortality. Second, the retrospective data lacked reliable and continuous adherence assessment indicators, which prevented quantification of the impact of treatment adherence on survival. This may lead to an underestimation of the survival benefit associated with good adherence. Third, due to the constraints of the retrospective data source, we were unable to account for certain potential confounders, including specific comorbidities and detailed lifestyle or behavioral factors. Their absence might influence the estimated associations. Furthermore, the lack of post-treatment longitudinal CD4^+^ T cell data in this study limits our conclusions to baseline measurements, which prevents an evaluation of how immune recovery dynamics may influence prognosis. In summary, while the mortality rate among ART-treated HIV/AIDS patients in Zhengzhou (2014–2024) remained low, risk factors including older age and lower baseline CD4^+^ T-cell count highlight opportunities for more targeted clinical management to further reduce AIDS-related mortality.

## 5 Conclusion

HIV-infected individuals receiving ART in Zhengzhou exhibit a high long-term survival rate. The main independent risk factors for survival prognosis are age >60 years and a baseline CD4^+^ T lymphocyte count < 50 cells/μL. Key measures to further extend the survival time of HIV-infected individuals include early identification and treatment of older adults and those with advanced immunodeficiency, along with strengthened prevention and management of opportunistic infections at ART initiation.

## Data Availability

The original contributions presented in the study are included in the article/Supplementary material, further inquiries can be directed to the corresponding author.
